# Performance Enhancement of Three-Dimensional MAPbI_3_ Perovskite Solar Cells by Doping Perovskite Films with CsPbX_3_ Quantum Dots

**DOI:** 10.3390/ma17061238

**Published:** 2024-03-07

**Authors:** Ming-Chen Tsai, Sheng-Yuan Chu, Po-Ching Kao

**Affiliations:** 1 Department of Electrical Engineering, National Cheng Kung University, Tainan 70101, Taiwan; janecat28@gmail.com; 2 Department of Electrophysics, National Chiayi University, Chiayi 60004, Taiwan; pckao@mail.ncyu.edu.tw

**Keywords:** perovskite, solar cell, perovskite quantum dot, heterogeneous nucleation centers

## Abstract

Perovskite thin films directly impact solar cell properties, making defect reduction crucial in perovskite solar cell research. In our study, we used perovskite quantum dots in the anti-solvent to act as nucleation centers in MAPbI3 thin films. These centers had lower nucleation barriers than homogeneous nucleation, improving perovskite crystallinity, reducing defects, and extending carrier lifetime. Fine-tuning the energy band also enhanced carrier transport. The most effective results were obtained using CsPb(Br_0.5_ I_0.5_)_3_ perovskite quantum dots. The resulting device, ITO/SnO_2_/MAPbI_3_ (300 nm)/spiro-OMeTAD (200 nm)/Ag (100 nm), achieved a 12.88% power conversion efficiency, a 16% increase from the standard element. The modified device maintained approximately 95% of its efficiency over 100 h in a 70% humidity environment.

## 1. Introduction

The need to increase low-carbon solar electricity production has become more urgent due to the necessity to mitigate climate change and address energy security concerns. Third-generation solar cells (SCs) are a promising avenue for renewable energy. They utilize semiconducting organic macromolecules [1], inorganic nanoparticles [2], or hybrids, and employ solution processing techniques. In particular, perovskite solar cells represent an emerging class of photovoltaic technology with distinct advantages. They are accessible, cost-effective, and have diverse structural variations. The synthesis methods for these materials are straightforward. Moreover, they exhibit remarkable tolerance for lattice defects, exceptional optoelectronic properties, and the ability to customize their energy bandgap by substituting different cations, metal centers, or halogens, covering the entire visible light spectrum.

Perovskite materials offer a wealth of benefits for various optoelectronic applications, including solar cells, light-emitting diodes, and light sensors. In 2009, the Kojima team in Japan pioneered the replacement of dyes in dye-sensitized solar cells with methylammonium lead iodide (CH_3_NH_3_PbI_3_) halide perovskite, achieving a remarkable photoelectric conversion efficiency of 3.8% [3]. Later, perovskite solar cells achieved remarkable efficiency levels, reaching 25.2%, placing them in competition with traditional silicon solar cells and representing a promising alternative. However, challenges like their susceptibility to photothermal instability, sensitivity to water and oxygen, and limited lifespan necessitate ongoing research and enhancements in perovskite materials. All solar cell types encounter non-radiative recombination losses, encompassing defect-assisted and Auger recombination, electron–phonon coupling, and band tail recombination. These losses result in deviations from ideal performance parameters for solar cells, including open-circuit voltage (Voc), short-circuit current density (Jsc), fill factor (FF), and photoelectric conversion efficiency (PCE). Furthermore, a high concentration of non-radiative recombination centers can facilitate the ingress of moisture and oxygen into the perovskite, hastening its degradation [4,5]. This presents a hurdle to the practical application and advancement of perovskite technology.

In order to give perovskite thin films better stability or crystallinity, many groups have recently invested in research related to multi-dimensional structure mixing. A recently successful strategy to improve the stability and water oxygen tolerance of perovskites is to mix 2D and 3D structures. It is well known that although 2D perovskites lag behind 3D perovskites in terms of performance due to poor carrier transport properties and mismatched energy bands, they are more stable to heat and moisture. For example, Lu et al. added ethylenediamine ammonium iodide (EDAI) to the three-dimensional MAPbI_3_ structure to improve PCE, and the MAPbI_3_-EDAI element with 0.8 mol% EDAI maintained about 75% of its initial performance after 72 h of illumination, while the original MAPbI_3_ loses 90% of its initial performance in just 15 h of runtime [6]. In addition, there are also some research groups dedicated to the development of related applications of 3D and 0D perovskite hybrids. Zhang et al. tuned the interfacial carrier dynamics by synthesizing uniform perovskite quantum dots CsPbX_3_ (X = mixed Br/I ions). The trap states on the perovskite surface and the perovskite grain boundaries can be effectively passivated, which promotes carrier separation and interface hole transport, reduces the carrier transition time, prolongs the lifetime, and improves the charge collection efficiency [7]. Huang et al. added CsPbI_3_ quantum dots synthesized by hot injection to the organic perovskite MAPbI_3_ to form a composite perovskite film. It was found that perovskite crystallization can be effectively enhanced by annealing at different temperatures, and the densest films can be obtained at an annealing temperature of 140 °C. The intensity of the preferred peak (110) of MAPbI_3_ is enhanced by increasing the crystal size and decreasing clustering [8]. P. Wang et al. introduced MAPbBr_3_ quantum dots into a solution-phase anti-solvent to act as nucleation centers and promote the growth of MAPbI_3_ films. Heterogeneous nucleation based on high lattice matching and low free energy significantly improves the crystallinity of MAPbI_3_ films, thereby prolonging the carrier lifetime and lower density of trap states in the films and increasing the photovoltaic properties and stability of solar cells [9]. However, the size and stability of the heterogeneous nucleation centers are critical to the crystallization of crystals, and there is no report on the effect of quantum dots with different anion ratios on MAPbI_3_ films. In this study, we adjust the ratio of bromide ions to iodide ions in CsPbX_3_ quantum dots and analyze the effect on MAPbI_3_ films.

## 2. Experimental Procedures

### 2.1. Synthesis of CsPb(Br/I)_3_ Quantum Dots

SnO_2_ colloid precursor (tin(IV) oxide 15% H_2_O colloidal dispersion), PbI_2_ (≥99.999, ultradry) and PbBr_2_ (≥99.998) and N,N-dymethylformammide (DMF), dimethylsulfoxide (DMSO), the methylammonium lead iodide (MAI_3_, 99.9%), methylammonium bromide (MaBr, ≥99%, anydrous) were obtained from USA Alfa Aesar (Ward Hill, MA, USA); CsI (≥99.999, anhydrous) and acetonitrile (ACN) were obtained from USA Acros Organics (Waltham, MA, USA); Spiro-OMETAD, Chlorobenzene (CB), ethyl acetate, Bis(trifluoromethane)sulfonimide lithium salt (Li-TFSI) were purchased from USA Sigma Aldrich (St. Louis, MO, USA). Ethyl acetate was purchased from Seedchem. I-Propyl alcohol (IPA, RS for HPLC- Isocratic grade), Acetone were purchased from Taiwan ECHO (Tainan City, Taiwan). All chemicals were used without further purification. We used 2 × 2 cm^2^ glass ITO substrates received from Luminescence Technology Corp, Taipei, Taiwan (15 Ω sq^−1^).

First, 0.3258 g of Cs_2_CO_3_, 16 mL of ODE, and 1 mL of OA were placed into a three-necked flask, heated to 120 °C using the oil bath method, and then heated for 1 h in a vacuum environment to remove water and oxygen from the three-necked flask. The vacuum environment in the bottle was changed to a nitrogen environment by feeding nitrogen to ensure that the reaction in the bottle was not affected by water and oxygen, and then the temperature was raised to 150 °C. When the solution was clarified, the Cs-OA solution was obtained.

Then, 1.08 mmol of PbX_2_ (X = Br, I) lead halide and 25 mL of ODE were placed into another three-necked flask, also heated to 120 °C, and heated for 1 h in a vacuum environment to remove water and oxygen from the container. Then, nitrogen was introduced to change the vacuum environment in the bottle to a nitrogen environment to ensure that the reaction in the bottle was not affected by water and oxygen. Following this, 2.5 mL OA and 2.5 mL OLA were injected with a syringe until the powder had completely dissolved; the temperature was raised to 170 °C and was maintained for 7 min to obtain a PbX_2_ solution.

Finally, 2 mL of the Cs-OA solution was sucked with a syringe and quickly injected into the PbX_2_ solution. After waiting for 5 s, the three-necked flask was immediately cooled via an ice bath method for 2 min to obtain a crude CsPb(Br/I)_3_ solution.

After synthesizing a crude solution of CsPb(Br/I)_3_ quantum dots, 30 mL of ethyl acetate was added to 15 mL of the crude solution and centrifuged at 5000 rpm for 3 min, and the supernatant was removed. The precipitate was then dispersed in 5 mL of toluene, and 7 mL of ethyl acetate was added, followed by centrifugation at 5000 rpm for 3 min, and the supernatant was removed. Finally, the precipitate was dispersed in 15 mL of toluene, and then centrifuged at 5000 rpm for 3 min, and the supernatant was collected to obtain a toluene solution with good dispersibility.

### 2.2. MAPbI_3_ Perovskite Solar Cell Fabrication Process

We used 180-nanometer-thick ITO-coated glass substrates with a sheet resistance of 15 Ω. Prior to depositing subsequent films, we sonicated the substrates in acetone, isopropyl alcohol, and deionized water for 15 min each. After drying them with a nitrogen gas flow, we exposed them to UV–ozone (Jelight UVO-42, Jelight Company, Inc., Irvine, CA, USA) for 15 min to remove any organic residues on the surface. The colloidal solution containing 15% SnO_2_ by weight was mixed with deionized water in a 1:1 ratio. The resulting solution was applied onto the ITO substrate by spin-coating at 4000 rpm for 30 s. To complete the preparation of the electron transport layer, the substrate was annealed for 40 min at 150 °C using a hotplate, adhering strictly to appropriate metrics and units. The perovskite precursor solution consisted of equimolar amounts of MAI (1M) and PbI_2_ (1M) dissolved in a DMF:DMSO mixture with a volume ratio of 4:1. The next step involved the uniform coating of the MAPbI_3_ precursor onto the SnO_2_ previously prepared. The two-step spin-coating process was conducted at 1500 rpm for 10 s and 5000 rpm for 20 s, with the addition of 200 µL chlorobenzene five seconds before the second step ended. After spin-coating, the sample underwent a 1 h annealing process at 100 °C to complete the perovskite layer preparation. The perovskite layers were also modified with CsPb(Br/I)_3_ quantum dots, with varying anion ratios (CsPbBr_3_/CsPb(Br_0.75_ I_0.25_)_3_/CsPb(Br_0.5_ I_0.5_)_3_/CsPb(Br_0.25_ I_0.75_)_3_/CsPbI_3_), added to chlorobenzene. The perovskite film was then crystallized, and a spiro-OMeTAD precursor solution was applied, consisting of 72.5 mg spiro-OMeTAD, 28.8 μL 4-tert-butylpyridine, and 17 fillerless units. Then, 5 μL of a solution of lithium bis (trifluoromethanesulfonyl)imide (Li-TFSI) in acetonitrile (520 mg Li-TFSI in 1 mL) was mixed with 1 mL of chlorobenzene. The resulting mixture was spin-coated at 4500 rpm for 30 s onto the perovskite film. Subsequently, a counter electrode made of Ag and 100 nm in thickness was deposited through thermal evaporation under a vacuum level of 1 × 10^−6^ Pa.

The film’s morphology was captured via a HITACHI SU8000 high-resolution scanning electron microscope (Tokyo, Japan), and the grain size statistics were attained by analyzing 50 grains utilizing the Image J software (https://imagej.net/, accessed on 4 March 2024). The surface roughness was measured with the Bruker Dimension Icon, while the XRD patterns were obtained through the Bruker AXS, Karlsruhe, Germany/D2 Phaser. X-ray photoelectron spectroscopy (XPS) was conducted with a PHI 5000 VersaProbe III (ULVAC-PHI. Inc., Chigasaki, Japan) for multiplexing purposes. PL and TRPL signals were obtained using an Edinburgh FS-5 spectrometer equipped with a 377 nm pulsed laser as the excitation source. The contact angle was measured with deionized water using an FTA-1000B (First Ten Angstroms, Portsmouth, VA, USA) instrument. J-V characteristics of the devices were measured under 1 sun solar spectrum illumination (AM 1.5 G) from an SS-X100R solar simulator with an intensity of 100 mW/cm^2^, which was calibrated using a standard silicon solar cell, with a Keithley 2400 parameter analyzer (USA Tektronix, Beaverton, OR, USA).

## 3. Results and Discussion

### 3.1. CsPb(Br/I)_3_ Quantum Dots HRTEM and Photoluminescence (PL) Spectroscopy

**Figure 1:**
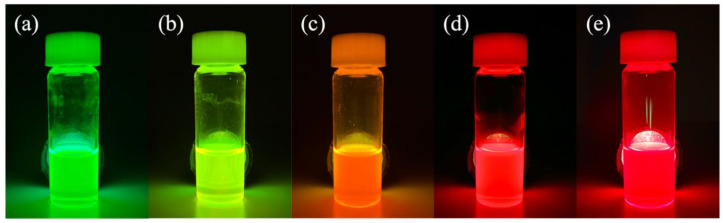
CsPb(Br/I)_3_ quantum dots with different anion ratios under the excitation of UV light with a wavelength of 365 nm: (**a**) CsPbBr_3_, (**b**) CsPb(Br_0.75_ I_0.25_)_3_, (**c**) CsPb(Br_0.5_ I_0.5_)_3_, (**d**) CsPb(Br_0.25_ I_0.75_)_3_, (**e**) CsPbI_3_.

First, we synthesized CsPb(Br/I)_3_ perovskite quantum dots with different ratios of Br and I. The synthesized CsPb(Br/I)_3_ perovskite quantum dots are CsPbBr_3_, CsPb(Br_0.75_ I_0.25_)_3_, CsPb(Br_0.5_ I_0.5_)_3_, CsPb(Br_0.25_ I_0.75_)_3_, CsPbI_3_, respectively. Figure 1 shows the synthesized quantum dots with different anion ratios under 365 nm ultraviolet light irradiation. The photoexcitation light presented by the quantum confinement effect can be seen initially. CsPbBr_3_ to CsPbI_3_ cover the range of visible light from green to red.

**Figure 2:**
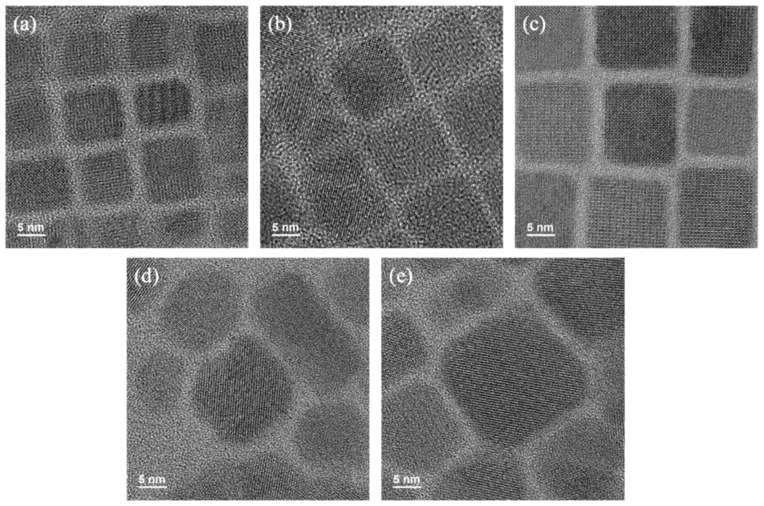
HRTEM images of CsPb(Br/I)_3_ quantum dots with different anion ratios: (**a**) CsPbBr_3_, (**b**) CsPb(Br_0.75_ I_0.25_)_3_, (**c**) CsPb(Br_0.5_ I_0.5_)_3_, (**d**) CsPb(Br_0.25_ I_0.75_)_3_, (**e**) CsPbI_3_.

**Figure 3:**
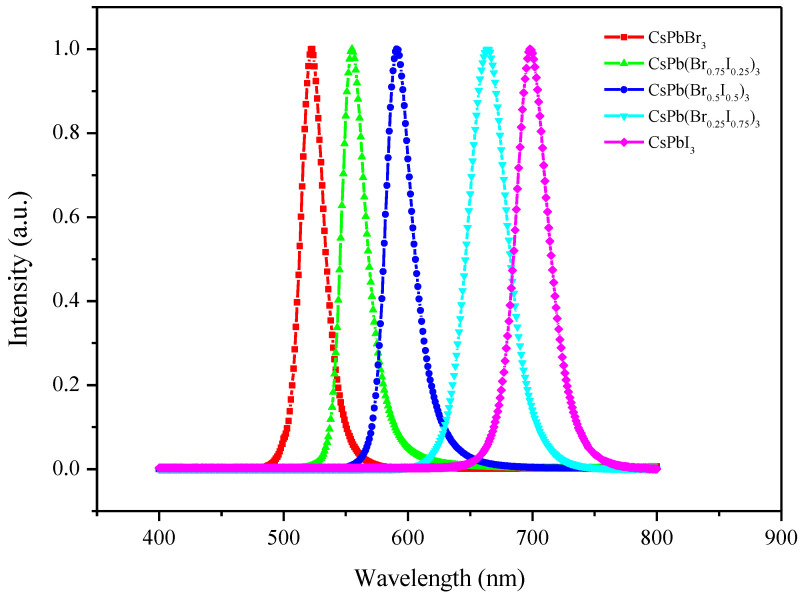
Photoluminescence spectroscopy of CsPb(Br/I)_3_ quantum dots with different anion ratios.

The surface morphology of the synthesized perovskite quantum dots can be understood by taking HRTEM images, as shown in Figure 2. It can be found that the quantum dot size of CsPbBr_3_ is the smallest, about 5–7 nm. As the proportion of I^−^ increases, the size of the quantum dots also increases gradually. This is because I^−^ has a larger ionic radius of 0.22 nm compared to the Br^−^ ionic radius of 0.196 nm. Increasing the doping ratio of iodine leads to lattice expansion and a red shift in the emission band, in accordance with the quantum confinement effect. The samples’ corresponding PL spectroscopy under 377 nm excitation is shown in Figure 3.

In addition, it can be observed that when the proportion of iodide ions in the halogen site of CsPb(Br/I)_3_ increases, the quantum dots change from square rows to polygons, and right angles degenerate into arcs. This is related to the instability of CsPbI_3_ compared with CsPbBr_3_.

### 3.2. SEM Analysis

**Figure 4:**
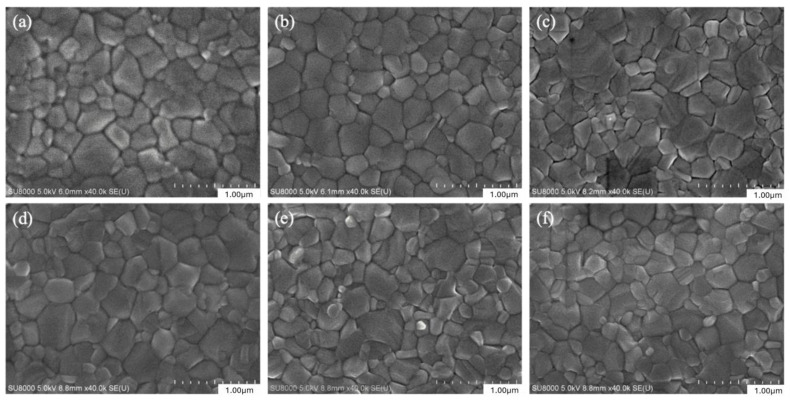
SEM of MAPbI_3_ perovskite films (**a**) without quantum dots and with CsPb(Br/I)_3_ quantum dots: (**b**) CsPbBr_3_, (**c**) CsPb(Br_0.75_ I_0.25_)_3,_ (**d**) CsPb(Br_0.5_ I_0.5_)_3_, (**e**) CsPb(Br_0.25_ I_0.75_)_3_, (**f**) CsPbI_3_.

**Figure 5:**
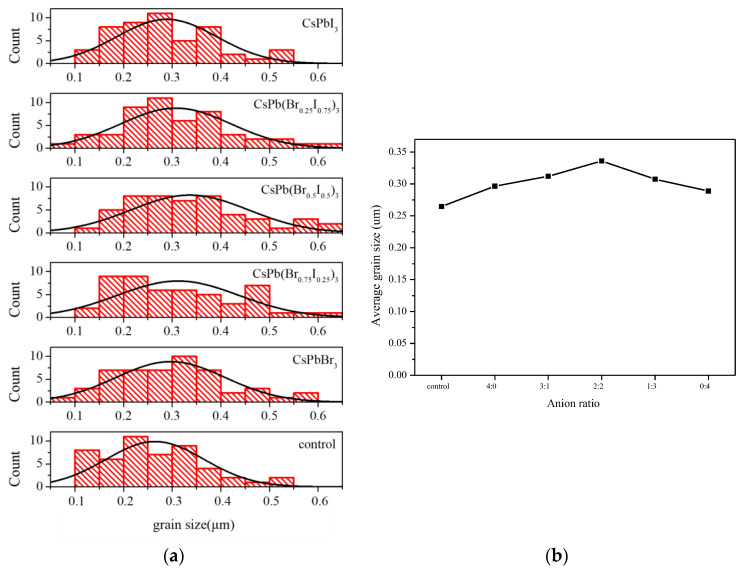
(**a**) Grain size of MAPbI_3_ perovskite without and with CsPb(Br/I)_3_ quantum dots; (**b**) average grain size of MAPbI_3_ perovskite without and with CsPb(Br/I)_3_ quantum dots.

First, CsPb(Br/I)_3_ quantum dots with different anion ratios (CsPbBr_3_, CsPb(Br_0.75_ I_0.25_)_3_, CsPb(Br_0.5_ I_0.5_)_3_, CsPb(Br_0.25_ I_0.75_)_3_, CsPbI_3_) were applied to MAPbI_3_ perovskite films, and the films were analyzed. The SEM top view of the MAPbI_3_ perovskite films added with different anion ratios of CsPb(Br/I)_3_ quantum dots was measured. Figure 4a–f show the surface morphology of MAPbI_3_ under the modification of CsPb(Br/I)_3_ quantum dots with different anion ratios. It can be observed that under the modification of CsPb(Br/I)_3_ quantum dots, the MAPbI_3_ films still maintain similar morphology. Further calculations demonstrated that the addition of quantum dots to CsPb(Br/I)_3_-modified MAPbI_3_ has a significant effect on the perovskite grain size. Specifically, the perovskite grains with quantum dots increased in size compared to those without quantum dots. Interestingly, the addition of CsPb(Br_0.5_ I_0.5_)_3_ resulted in the most substantial increase in the average grain size, as indicated in Figure 5a,b, from 265 nm to 336 nm. Increasing the size of the grains can decrease the number of grain boundaries, leading to improved device efficiency as grain boundaries can cause charge trapping. As demonstrated by previous literature, perovskite quantum dots added to anti-solvents can effectively act as heterogeneous nucleation centers [9]. Because heterogeneous nucleation has a lower nucleation free-energy barrier than homogeneous nucleation [10], 3D perovskites tend to grow around quantum dots, and the formation of 3D perovskite grains become easier. The process of heterogeneous nucleation is related to the diameter of the seed crystal. To be an effective crystallization catalyst, the seed particles need to exceed a defined minimum size. When the seed crystal becomes larger, the free-energy barrier decreases. In other words, the film has lower nucleation activation energy and a faster nucleation rate [11]. Therefore, the addition of CsPb(Br/I)_3_ quantum dots is favorable for 3D MAPbI_3_ perovskite crystallization. In addition, it can be observed that with the increase in iodide ions in the quantum dots, the addition of the quantum dots CsPb(Br_0.5_ I_0.5_)_3_ will have the best crystallinity. If the proportion of iodide ions continues to increase, the perovskite grain size will tend to decrease slightly. It is speculated that the size of the quantum dots increases with the increase in iodide ions in the quantum dots, thereby reducing the free-energy barrier that needs to be exceeded during the crystallization of the 3D perovskite, which is favorable for crystal formation. However, when the size of the quantum dots continues to increase, it will lead to the free-energy barrier being too low, which makes the perovskite crystallize too quickly, resulting in uneven film growth and the formation of defects.

### 3.3. AFM Analysis

**Figure 6:**
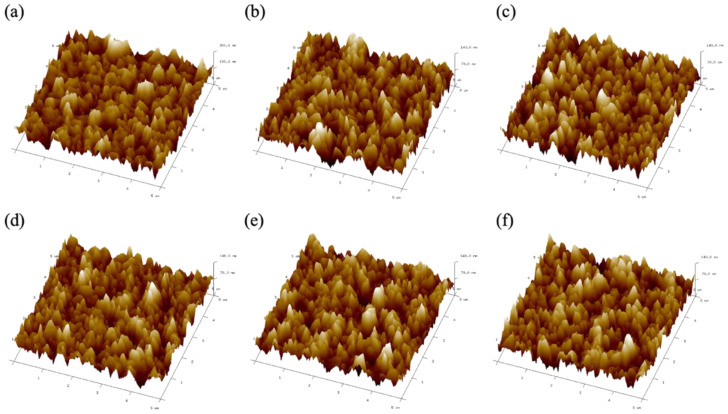
AFM surface scan of MAPbI_3_ perovskite without and with CsPb(Br/I)_3_ quantum dots: (**a**) without QDs, and with (**b**) CsPbBr_3_, (**c**) CsPb(Br_0.75_ I_0.25_)_3_, (**d**) CsPb(Br_0.5_ I_0.5_)_3_, (**e**) CsPb(Br_0.25_ I_0.75_)_3_, and (**f**) CsPbI_3_.

In order to study the effect of CsPb(Br/I)_3_ quantum dots with different anion ratios on the flatness of perovskite films, we compared the results of the AFM measurements, as shown in Figure 6a–f. The average roughness (Ra) and root-mean-square roughness (Rms) of the perovskite films with CsPb(Br/I)_3_ quantum dots with different anion ratios were measured, as shown in Table 1. The measurement results show that the 3D perovskite films with CsPb(Br/I)_3_ quantum dots have better surface flatness than the pristine MAPbI_3_ films. When the quantum dots are CsPb(Br_0.5_ I_0.5_)_3_, the performance is the best, and the root-mean-square roughness is 17.4 nm, which is about 7 nm different from the pristine films. This result is consistent with the grain size results measured by SEM in the previous section, since a large and uniform crystal size reduces grain boundaries and makes the film flatter.

### 3.4. XRD Analysis

**Figure 7:**
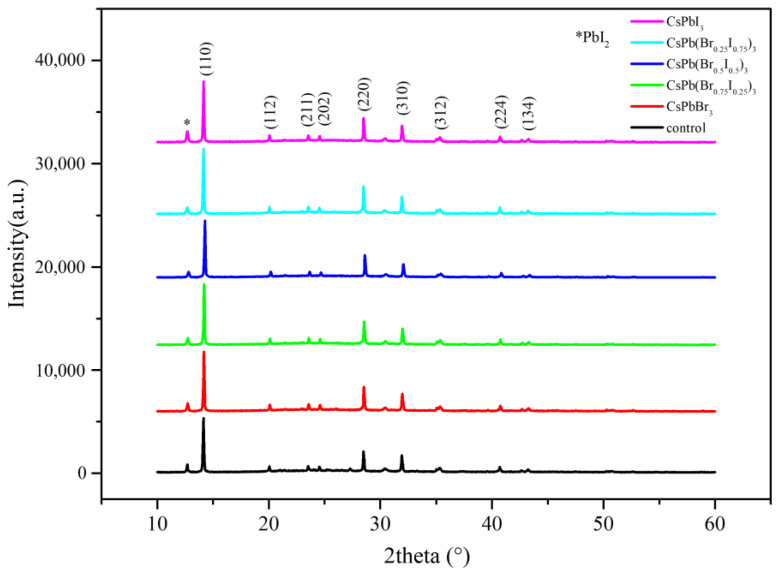
XRD patterns of MAPbI_3_ perovskite without or with CsPb(Br/I)_3_.

The crystal structure of the perovskite thin film was analyzed by X-ray diffraction (XRD). It can be seen from Figure 7 that the perovskite films of all parameters have diffraction peaks at 14.15°, 20.05°, 23.53°, 24.52°, 28.49°, 31.93°, 35.02°, 40.71°, and 43.25°, which correspond to (110), (112), (211), (202), (220), (310), (312), (224), and (134) for the cubic phase of MAPbI_3_ perovskite, respectively. Compared with the pristine film, the perovskite films with different anion ratio quantum dots have no obvious phase shift or phase transition, and no new diffraction peaks appear. It is shown that the crystal structure of perovskite films with CsPb(Br/I)_3_ quantum dots is unchanged. In addition, we know that PbI_2_ is the precursor of MAPbI_3_: the diffraction peak at 12.6° representing the PbI_2_ (001) lattice plane is from the incomplete conversion of the initial precursor, and in calculating the ratio of PbI_2_ (001) to MAPbI_3_ (110), the largest value can be observed for the perovskite film with CsPb(Br_0.5_ I_0.5_)_3_ quantum dots, as shown in Table 2. This proves that selecting quantum dots of appropriate size as heterogeneous nucleation centers can make perovskite crystallization easier, and the reaction during the formation of perovskite films is more complete. In addition, comparing the peak intensity ratios of (110)/(310) among the parameters, it can be found that the perovskite film with CsPb(Br_0.5_ I_0.5_)_3_ quantum dots has the highest value, as shown in Table 2. This indicates that the CsPb(Br_0.5_ I_0.5_)_3_ quantum dots promote the growth of perovskite crystals and have a preferential orientation of the (110) plane, thereby improving the crystallinity of perovskite films. This is because after adding CsPb(Br/I)_3_ QDs to the MAPbI_3_ perovskite films as heterogeneous nucleation centers, MAPbI_3_ is more inclined to nucleate and grow along the crystal planes of CsPb(Br/I)_3_ QDs. Since CsPb(Br/I)_3_ QDs have a (110) crystal plane, and MAPbI_3_ has a similar crystal lattice, perovskite films are easier to grow along this crystal plane [12]. In addition, it is concluded that it may also be related to the stability of quantum dots. The stability decreases due to an increase in the proportion of iodide ions. The instability of quantum dots may cause perovskite crystallization to be affected and the possibility of causing defects to increase.

### 3.5. Absorption Spectrum

**Figure 8:**
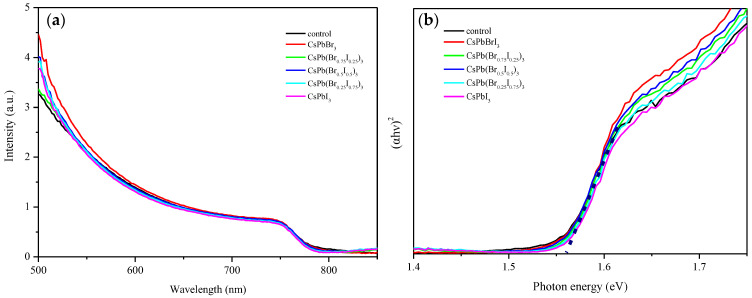
(**a**) Absorption spectra of MAPbI_3_ perovskite without or with CsPb(Br/I)_3_ quantum dots; (**b**) Tauc plot converted from absorption spectra. Dashed lines mark the corresponding positions of the tangents to the band gap.

Next, the optoelectronic properties of the MAPbI_3_ perovskite layer were measured and analyzed. First, the absorption spectrum was measured to compare the intensity and wavelength change of the light absorption of the MAPbI_3_ perovskite films with and without CsPb(Br/I)_3_ quantum dots, and the energy band structure was analyzed. Figure 8a shows the absorption spectra of the pristine MAPbI_3_ perovskite film and the perovskite films with different anion ratios of CsPb(Br/I)_3_ quantum dots at wavelengths of 500 nm–800 nm. It can be observed in the visible red band that the absorption curves of perovskite films with different anion ratios of CsPb(Br/I)_3_ quantum dots and the pristine MAPbI_3_ perovskite film almost overlap. This means that the addition of CsPb(Br/I)_3_ quantum dots has little effect on the absorptivity of perovskite films.

In addition, the energy bandgap was analyzed by converting the obtained absorption spectrum into a Tauc plot. Tauc plots are mainly used to determine the energy bandgap of semiconductors. The formula is as follows:

$${\left(\alpha h\nu \right)}^{1/n}=A(h\nu -E_{g})$$

where *α* is the absorption coefficient of the material, *hν* is the photon energy, *A* is a constant, and *E_g_* is the semiconductor energy bandgap. The abscissa of the Tauc plot is *hν*, and the ordinate is ${\left(\alpha h\nu \right)}^{1/n}$. Extending the linear region in the diagram to the intersection with the abscissa is the energy bandgap of the material. Since the perovskite material is a direct-bandgap semiconductor material, the Tauc plot can be drawn when n is 1/2, as shown in Figure 8b. Because the absorption curves of MAPbI_3_ films with different halogen ratios of CsPb(Br/I)_3_ quantum dots are almost identical to the curve of pristine MAPbI_3_ perovskite film, the Tauc plots are also very similar. It can be seen from Figure 8b that the energy bandgaps of all of the perovskite films are 1.56 eV.

### 3.6. UPS and Energy Band Analysis

**Figure 9:**
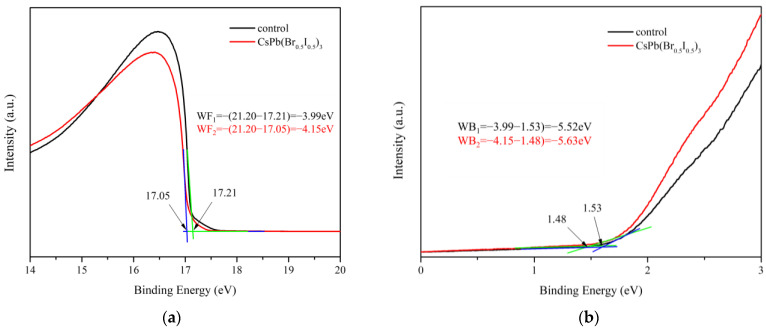
(**a**,**b**) UPS energy spectrum of MAPbI_3_ perovskite without and with CsPb(Br_0.5_ I_0.5_)_3_ quantum dots.

**Figure 10:**
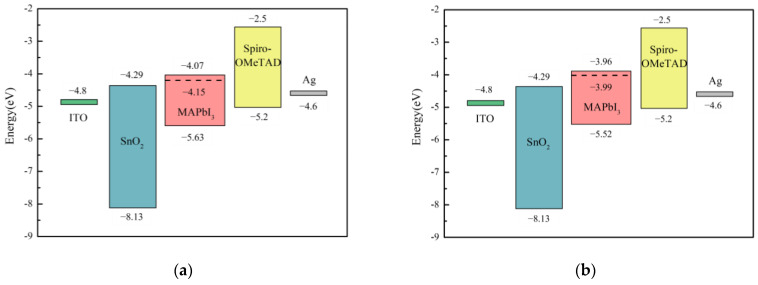
(**a**) Energy band diagram of solar cell without quantum dots added to MAPbI_3_ perovskite layer; (**b**) energy band diagram of solar cell with CsPb(Br_0.5_ I_0.5_)_3_ quantum dots added to MAPbI_3_ perovskite layer. Dashed lines are used to indicate the relative positions of the public functions.

Figure 9a presents the binding energy positions corresponding to the secondary electron cut-off potentials of the pristine MAPbI_3_ perovskite film and the MAPbI_3_ perovskite film with CsPb(Br_0.5_ I_0.5_)_3_ quantum dots. The calculation showed that work function of the perovskite film modified with quantum dots CsPb(Br_0.5_ I_0.5_)_3_ changed from −3.99 eV to −4.15 eV. In addition, the valence band of the pristine MAPbI_3_ perovskite and the perovskite film with CsPb(Br_0.5_ I_0.5_)_3_ quantum dots can be calculated from Figure 9b. The conduction band of the perovskite layer can be calculated from the energy bandgap obtained from the Tauc plot. The conduction band of the perovskite film changed from −3.96 eV to −4.07 eV when CsPb(Br_0.5_ I_0.5_)_3_ quantum dots were added. Then, we obtained the energy band of the MAPbI_3_ perovskite, as shown in Figure 10.

The results show that the pristine perovskite film is n-type because its Fermi level (E_F_) is close to the conduction band. In a previous study, it was demonstrated that perovskite crystals are biased towards n-type semiconductors due to donor-type surface states (Pb^0^) [13]. When CsPb(Br_0.5_ I_0.5_)_3_ quantum dots were added to the perovskite film, the Fermi level moved downward. This means that the surface state of MAPbI_3_ is reduced, the n-type characteristic is weakened, and the transfer of holes between the perovskite layer and the hole transport layer is more favorable. In addition, the energy difference between the conduction band of SnO_2_ and the perovskite film improved by CsPb(Br_0.5_ I_0.5_)_3_ quantum dots is reduced, so that the electron transfer efficiency is higher in the perovskite element. Reducing the energy barrier of charge transfer at the interface can effectively reduce the Voc loss of the element [14].

### 3.7. Photoluminescence Spectrum (PL)

**Figure 11:**
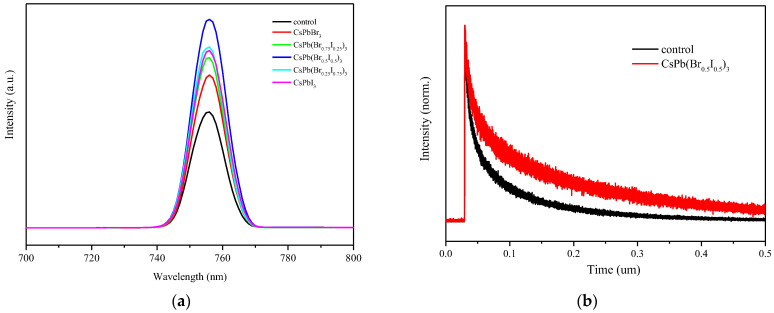
(**a**) PL spectra of MAPbI_3_ perovskite without or with CsPb(Br/I)_3_ quantum dots; (**b**) TRPL spectra of MAPbI_3_ perovskite with or without CsPb(Br_0.5_ I_0.5_)_3_ quantum dots.

The energy transfer process of photoexcited carriers was analyzed using a photoluminescence spectrum. The PL measurement results in Figure 11a show that the emission peaks of the pristine MAPbI_3_ perovskite film and the perovskite films with different anion ratios of CsPb(Br/I)_3_ quantum dots are all 756 nm. This indicates that the addition of quantum dots CsPb(Br/I)_3_ to the MAPbI_3_ perovskite films does not cause any shift (blue shift or red shift) in its emission spectrum, which is consistent with the absorption spectrum.

In the emission intensity, it can be observed that when the proportion of iodide ions in CsPb(Br/I)_3_ quantum dots increases, the luminescence intensity of perovskite films gradually increases, reaching the highest value when the added quantum dots are CsPb(Br_0.5_ I_0.5_)_3_. However, the emission intensity of the perovskite films with quantum dots CsPb(Br_0.25_ I_0.75_)_3_ and CsPbI_3_ begins to weaken. In addition, the PL intensities of the perovskite films with CsPb(Br/I)_3_ quantum dots are all higher than the pristine MAPbI_3_ perovskite films.

This result is positively correlated with the surface morphology and surface roughness of perovskite films. It can be seen from the previous measurement results that the average grain size is the largest and the distribution is most uniform when CsPb(Br_0.5_ I_0.5_)_3_ quantum dots are added. Elevated surface defects within the film lead to the creation of recombination centers bridging the conduction and valence bands. This escalation in recombination centers heightens the likelihood of charge quenching and non-radiative recombination, subsequently impacting the photoluminescence intensity.

### 3.8. TRPL Analysis

To explore the photon lifetime of the perovskite material, a TRPL measurement was performed to obtain the PL decay time of the perovskite layer. Figure 11b compares the emission decay curves of the original MAPbI_3_ perovskite film and the perovskite film modified with CsPb(Br_0.5_ I_0.5_)_3_ quantum dots. First, the sample was excited with a laser pulse light source with a wavelength of 377 nm, and then the measured curve was fit using a biexponential decay function. The formula is as follows:

$$F\left(t\right)=A_{1}\exp\left(\frac{-t}{\tau_{1}}\right)+A_{2}\exp\left(\frac{-t}{\tau_{2}}\right)+y_{0}$$

In this equation, $A_{1}$ and $A_{2}$ represent the decay amplitudes, $\tau_{1}$ denotes the fast decay lifetime, $\tau_{2}$ signifies the slow decay lifetime, and $y_{0}$ stands for a constant responsible for the baseline shift [15]. As per the analysis of decay curves in previous literature pertaining to perovskite materials, the fast decay component, $\tau_{1}$, can be attributed to free carriers generated through direct radiative recombination between carriers in the conduction and valence bands. On the other hand, the slow decay component, $\tau_{2}$, is associated with trap-assisted recombination induced by defects [16,17].

The fitting data of this experiment are shown in Table 3. From the fitting results, the $\tau_{1}$ of the MAPbI_3_ perovskite film doped with CsPb(Br_0.5_ I_0.5_)_3_ quantum dots increased from 22.7 ns to 29.1 ns. It is proved that the surface defect density of perovskite films modified by CsPb(Br_0.5_ I_0.5_)_3_ quantum dots is reduced. The $\tau_{2}$ increased from 133.9 ns to 241.4 ns, indicating that the crystallinity of the film was better, and the defects were reduced. The average carrier lifetime $\tau_{avg}$ increased from 106.4 ns to 218.5 ns, indicating that the non-radiative recombination of the film decreased, and the PL carrier lifetime increased.

### 3.9. I-V/PCE and Lifetime Measurement

**Figure 12:**
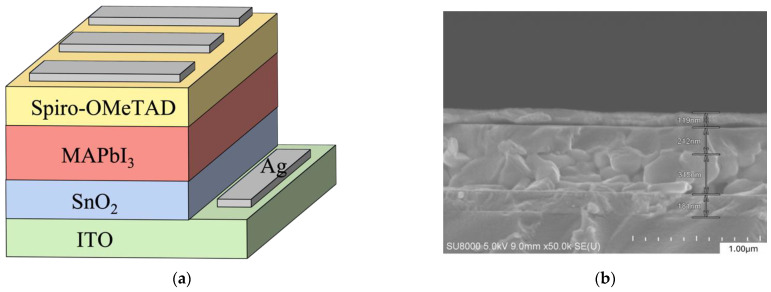

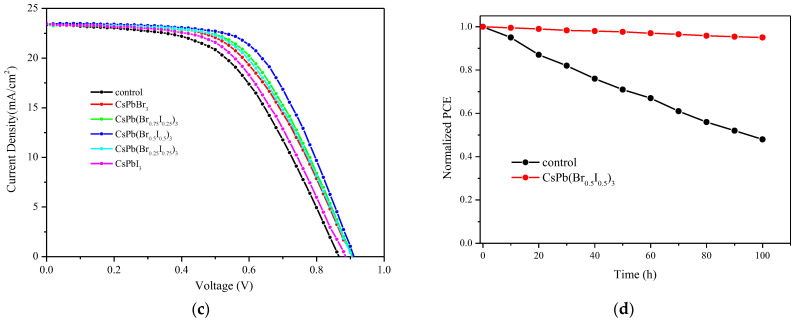
(**a**) Schematic diagram of solar cell element structure; (**b**) SEM cross-section of perovskite solar cell; (**c**) J-V characteristic curves of solar cells with and without CsPb(Br/I)_3_ quantum dots; (**d**) normalized PCE as a function of time for perovskite solar cells without and with CsPb(Br_0.5_I_0.5_)_3_ quantum dots.

The perovskite films without and with different anion ratios of CsPb(Br/I)_3_ quantum dots (CsPbBr_3_/CsPb(Br_0.75_ I_0.25_)_3_/CsPb(Br_0.5_ I_0.5_)_3_/CsPb(Br_0.25_ I_0.75_)_3_/CsPbI_3_) were fabricated into solar cells with the structure of ITO/SnO_2_/MAPbI_3_ (300 nm)/spiro-OMeTAD (200 nm)/Ag (100 nm), and the electrical properties were measured and analyzed. The component structure shown in Figure 12a,b is a SEM cross-sectional view of the solar cell structure. Figure 12c is the characteristic curve of the scanning voltage corresponding to the current density (J-V). The main photovoltaic parameters are shown in Table 4. It can be observed that the photoelectric conversion efficiency (PCE) of all of the solar cells modified with CsPb(Br/I)_3_ quantum dots is higher than the control element. When quantum dots are used as CsPb(Br_0.5_ I_0.5_)_3_, the best results are obtained, and the PCE reaches 12.88%. Compared with standard components, the efficiency is increased by about 16%. In addition, as shown in Table 4, the short-circuit current (Jsc) does not change much between the different parameters, which are all around 23.4 mA/cm^2^, while the open-circuit voltage (Voc) and fill factor (FF) exhibit good improvement. Interface engineering is crucial for the improvement in the open-circuit voltage and fill factor [18]. The loss of open-circuit voltage is mainly due to non-radiative recombination caused by deep defects [19]. In addition to non-radiative recombination losses, the fill factor is also affected by charge transport problems. Due to the addition of CsPb(Br/I)_3_ quantum dots, both the bulk and surface carrier recombination of the perovskite film are suppressed, so the V_OC_ is improved.

Furthermore, when CsPb(Br/I)_3_ quantum dots are introduced, the carrier transport is enhanced due to the reduced grain boundaries, and the mobility along the preferential (110) orientation is greater than the mobility along the other directions [20] and favorable energy band alignment, thus increasing the corresponding fill factor. In addition, the difference in element characteristics between CsPb(Br/I)_3_ quantum dots with different anion ratios is presumed to be mainly due to the difference in the size and stability of the quantum dots. The seed size in a heterogeneous nucleation system affects the energy and crystallization rate required for crystallization. Therefore, selecting an appropriately sized seed crystal is very important for the process of heterogeneous nucleation. If the CsPb(Br/I)_3_ quantum dots used as seeds are not stable, they may degrade and cause defects in perovskite film. In addition, the standard element and the element modified with CsPb(Br_0.5_ I_0.5_)_3_ quantum dots were placed in an environment with a relative humidity of 70% for 100 h to measure the PCE change. It can be found that at the 100th hour of the element, the standard element PCE drops by 50%, while the improved element maintains at about 95%, as shown in Figure 12d. This is because the reduced grain boundaries result in denser films, and atmospheric moisture is less likely to damage the organic perovskite films through defects.

## 4. Conclusions

This study delves into the enhancement of MAPbI_3_ perovskite films through the introduction of CsPb(Br/I)_3_ quantum dots. The investigation encompasses an analysis of the perovskite layer’s morphology, crystallinity, defect mitigation, and carrier lifetime, with a subsequent evaluation of the modified perovskite material’s performance in solar cell applications for photoelectric conversion. The outcomes reveal the efficacy of perovskite quantum dots as heterogeneous nucleation centers. This efficacy arises from their ability to facilitate the formation of perovskite grains with lower nucleation free-energy barriers compared to homogeneous nucleation. Moreover, the preferential orientation of crystal planes contributes to improved crystallinity, reduced defects, and prolonged carrier lifetimes. Furthermore, the energy band adjustment enhances carrier transport. Nonetheless, the size and stability of the quantum dots play pivotal roles in the heterogeneous nucleation process. Therefore, the careful selection of suitable quantum dots as nucleation centers is essential.

The study explores the efficiency and longevity of perovskite solar cells enhanced with quantum dots featuring different anion ratios. The results demonstrate that CsPb(Br_0.5_ I_0.5_)_3_ quantum dots yield the most substantial improvement, achieving a power conversion efficiency (PCE) of 12.88%. This represents a remarkable 16% increase compared to the control device. Additionally, when subjected to a 70% humidity environment for 100 h, the PCE of the CsPb(Br_0.5_ I_0.5_)_3_-enhanced device only dropped to 95% of its original value, while the control device experienced a more significant decline to 50% of its initial performance.

## Figures and Tables

**Table 1:** Surface roughness measurement results.

Sample	Ra (nm)	Rms (nm)
control	18.9	24.4
CsPbBr_3_	14.5	18.4
CsPb(Br_0.75_ I_0.25_)_3_	15.0	18.9
CsPb(Br_0.5_ I_0.5_)_3_	13.8	17.4
CsPb(Br_0.25_ I_0.75_)_3_	15.8	19.9
CsPbI_3_	15.6	19.7

**Table 2:** XRD crystal-phase analysis of MAPbI3 perovskite without and with CsPb(Br/I)_3_ quantum dots.

Sample	MAPbI_3_(110)	PbI_2_(001)	MAPbI_3_(310)	(110)/(310)	MAPbI_3_(110)/ PbI_2_(001)
control	5358	858	1741	3.08	6.245
CsPbBr_3_	5503	868	1770	3.11	6.34
CsPb(Br_0.75_ I_0.25_)_3_	5976	767	1655	3.61	7.791
CsPb(Br_0.5_ I_0.5_)_3_	5568	605	1346	4.14	9.203
CsPb(Br_0.25_ I_0.75_)_3_	6399	714	1782	3.59	8.962
CsPbI_3_	5987	1147	1675	3.57	5.22

**Table 3:** Comparison of various parameters of TRPL.

Sample	A_1_	$\tau_{1}$(ns)	A_2_	$\tau_{2}$(ns)	$\tau_{avg}$
control	0.83	22.7	0.43	133.9	106.4
CsPb(Br_0.5_I_0.5_)_3_	0.27	29.1	0.27	241.4	218.5

**Table 4:** Photovoltaic parameters of perovskite solar cells without and with CsPb(Br/I)_3_ quantum dots.

Sample	Jsc (mA/cm^2^)	Voc (V)	FF (%)	PCE (%)
control	23.46	0.876	53.65	11.03
CsPbBr_3_	23.48	0.907	0.55	11.64
CsPb(Br_0.75_ I_0.25_)_3_	23.34	0.908	0.57	12.14
CsPb(Br_0.5_ I_0.5_)_3_	23.48	0.915	0.60	12.88
CsPb(Br_0.25_ I_0.75_)_3_	23.45	0.905	0.56	11.91
CsPbI_3_	23.46	0.886	0.54	11.15

## Data Availability

Data are contained within the article.
